# Association of BNT162b2 Vaccine Third Dose Receipt With Incidence of SARS-CoV-2 Infection, COVID-19–Related Hospitalization, and Death Among Residents of Long-term Care Facilities, August to October 2021

**DOI:** 10.1001/jamanetworkopen.2022.19940

**Published:** 2022-07-01

**Authors:** Khitam Muhsen, Nimrod Maimon, Amiel Yaron Mizrahi, Baruch Varticovschi, Omri Bodenheimer, Dani Cohen, Ron Dagan

**Affiliations:** 1 Department of Epidemiology and Preventive Medicine, School of Public Health, the Sackler Faculty of Medicine, Tel Aviv University, Ramat Aviv, Tel Aviv, 6139001, Israel; 2 Israel Ministry of Health, Senior Shield Project, Airport City, Israel; 3 Faculty of Health Sciences, Ben-Gurion University of the Negev, Beer-Sheva, Israel; 4 Soroka University Medical Center, Beer-Sheva, Israel

## Abstract

**Importance:**

COVID-19 vaccine might be less immunogenic and effective among residents of long-term care facilities (LTCFs).

**Objective:**

To examine the association of BNT162b2 third dose (first booster dose) with overall SARS-CoV-2 infection, COVID-19 hospitalizations, and mortality among LTCF residents during a nationwide surge of the Delta variant in Israel.

**Design, Setting, and Participants:**

This observational cohort study conducted nationwide COVID-19 surveillance in LTCFs in Israel between August and October 2021. Participants were residents of LTCFs aged 60 years or older.

**Exposures:**

Vaccination with the third dose of BNT162b2 vaccine vs receipt of 2 doses at least 5 months earlier, based on self-preference and choice.

**Main Outcomes and Measures:**

The cumulative incidences of reverse transcription–polymerase chain reaction (RT-PCR)–confirmed SARS-CoV-2 infection, COVID-19 hospitalizations, and COVID-19–related deaths more than 7 days after vaccination with the third dose were compared between the groups using Kaplan-Meier curves. Hazard ratios (HRs) and 95% CIs were obtained using multivariable Cox regression models.

**Results:**

Among 18 611 residents included in the analysis, 12 715 (68.3%) were female, 463 (2.5%) were from the Arab population, 16 976 (91.2%) were from the general Jewish population, and 618 (3.3%) were from the ultraorthodox Jewish population; the mean (SD) age was 81.1 (9.2) years; 16 082 residents received their first booster dose (third dose) and 2529 were vaccinated with 2 doses at least 5 months earlier. The median (IQR) follow-up durations were 66 (60-70) days among 3-dose recipients and 56 (53-62) days among 2-dose–only recipients; 107 residents had SARS-CoV-2 infection after 7 days following vaccination with the booster dose compared with 185 among the 2-dose only group (cumulative incidence: 0.7% vs 7.5%; adjusted HR, 0.11 [95% CI, 0.07-0.15]). The respective adjusted HRs were 0.07 (95% CI, 0.03-0.14) and 0.10 (95% CI, 0.04-0.24) for the associations of vaccination with the third dose with hospitalization for mild-to-moderate COVID-19 and severe illness. Five COVID-19-related deaths occurred among the third dose vaccinees during the follow-up period compared with 22 among the 2-dose–only vaccinees (cumulative rate: 0.04% vs 0.9%; adjusted HR, 0.04 [95% CI, 0.009-0.16]).

**Conclusions and Relevance:**

This cohort study found significant inverse associations between vaccination with the third dose of the BNT162b2 vaccine with overall SARS-CoV-2 infection, COVID-19 hospitalizations, severe disease, and COVID-19–related deaths among LTCF residents during a massive surge caused by the Delta variant in Israel.

## Key Points

**Question:**

What is the association between vaccination with the third dose of BNT162b2 vaccine and the incidence of COVID-19 among residents of long-term care facilities?

**Findings:**

In this cohort study of 18 611 residents at 640 long-term care facilities, the risk of SARS-CoV-2 infection, COVID-19–related hospitalizations, and COVID-19–related deaths was 89% to 96% lower among residents who received the third dose of BNT162b2 vaccine compared with vaccinees who received 2 doses at least 5 months earlier during the Delta variant surge in Israel.

**Meaning:**

The third dose of BNT162b2 vaccine was associated with a reduced burden of SARS-CoV-2 infection, COVID-19–related hospitalizations, and COVID-19–related deaths in long-term care facilities.

## Teaser

This cohort study investigates the association of the third dose of BNT162b2 vaccine with overall SARS-CoV-2 infection, COVID-19 hospitalization, and mortality among long-term care facility residents during the Delta variant surge in Israel.

## Introduction

In Israel, the BNT162b2 (Pfizer-BioNTech) mRNA COVID-19 vaccination campaign, initiated on December 2021, successfully interrupted the COVID-19 epidemic.^1,2,3^ This campaign was estimated to avert 158 665 SARS-CoV-2 infections, 24 597 COVID-19 hospitalizations, and 5532 deaths, with 91.0% of deaths averted among persons aged 65 years and older.^3^ The incidence of reverse transcription–polymerase chain reaction (RT-PCR)–confirmed SARS-CoV-2 infection dropped from 9200 daily cases in mid-January to 14 daily cases by end of May 2021.^4^ In June 2021, a new surge in COVID-19 occurred, including among fully vaccinated individuals who received 2 doses.^4,5,6^ Among persons aged 60 years or older who became fully vaccinated with 2 doses in January 2021, the risk of infection was increased by 1.6-fold compared with those vaccinated in March 2021,^5^ suggesting waning immunity, which was also evident in younger ages. This surge was caused by the Delta variant of concern, which rapidly spread in Israel and became dominant until December 2021, when it was replaced by the Omicron variant.^7^

Older individuals who reside in long-term care facilities (LTCFs) are among the most at risk for severe and fatal COVID-19.^8,9,10,11,12^ Individuals in this population often experience multiple comorbidities, have difficulty appropriately applying barriers to infection,^13,14,15^ and have low immune responses and accelerated decline in immunity following immunization with BNT162b2 vaccine.^15^ Moreover, most LTCFs are overcrowded and poorly prepared to implement infection control policies owing to a shortage of staff, personal protective equipment, and the low level of training for health care workers.^16,17^ Therefore, in mid-April 2020, Israel established a dedicated national level task force with the aim of protecting the LTCF sector from the spread of SARS-CoV-2. This task force, Senior Shield, (Magen Avot v’Imahot),^9,18^ is a government agency tasked to support the ministerial organizations and LTCFs in managing the COVID-19 epidemic, and included all LTCFs in Israel.^9,17,18^ The program included routine weekly SARS-CoV-2 RT-PCR screening tests on all LTCF personnel. Additionally, the Senior Shield task force was responsible for vaccinating all health care workers and residents of LTCFs, including vaccination with a third BNT162b2 dose in August 2021.

We reported a significant reduction in COVID-19 incidence and hospitalizations among residents of LTCFs following the introduction of the first booster dose (ie, third dose) vaccination.^19^ However, so far, understanding the individual-level association between vaccination with the third dose of BNT162b2 vaccine and various outcomes among the high-risk, older residents of LTCFs is limited. The aim of this study was to examine the association of the BNT162b2 first booster dose (ie, third dose) with RT-PCR–confirmed SARS-CoV-2 infection, COVID-19 hospitalizations, and mortality among LTCF residents.

## Methods

### Study Design

This cohort study was undertaken as part of Senior Shield routine SARS-CoV-2 infection surveillance in LTCFs. This report followed the Strengthening the Reporting of Observational Studies in Epidemiology (STROBE) reporting guideline.

### Ethics Approval

The study protocol was approved by the institutional review board of the Soroka University Medical Center, Beer-Sheva, Israel. An exempt from written informed consent was given due to the retrospective study design.

### Study Population

Senior Shield serves all LTCFs in Israel. The LTCFs vary in bed size, funding sources and the characteristics of the resident populations. This includes state-funded chronic geriatric facilities, a few acute geriatric care facilities (eg, rehabilitation services, mechanical ventilation) funded by the sick funds, welfare facilities, and others.

All Israeli citizens have universal health insurance because of the National Health Insurance Law. COVID-19 tests, vaccines, and hospitalizations were provided at no cost to citizens. Senior Shield was established to support the prevention and control of COVID-19 in LTCFs, via providing personal protective equipment, screening for the early detection of infected health care workers and residents, vaccination, and medical care. These activities are performed using standardized protocols across all facilities and not related to vaccination status.

On July 30, 2021, Israel authorities approved the administration of the first booster (third dose) to all individuals aged 60 years or older who had received a second dose of BNT162b2 at least 5 months earlier.^20,21^ On August 1, 2021, Senior Shield task force began the first booster dose campaign in LTCFs, by designated teams in an organized and synchronized manner, and by August 22, most residents were already vaccinated with the third dose. The vaccination campaign was undertaken in collaboration with Magen David Adom (MDA), Israel's national emergency medical services organization. MDA has countrywide stations, well-trained medics, vehicles (including for transport in cool conditions), and experience in leading national operations. MDA central office received from Senior Shield headquarters a list of residents and LTCFs who should be vaccinated. MDA medics obtained the vaccines from a central warehouse and transported them to the target LTCFs, where both residents and health care workers were vaccinated. The medics documented the vaccination into a central database, through which data were transferred to the Senior Shield database. The study groups included residents who received the third dose (third-dose group) of BNT162b2 vaccine, and the comparison group included residents for whom at least 5 months had elapsed since dose 2. We included individuals who did not have a documented SARS-CoV-2 infection until the starting follow-up date. Individuals who were previously infected with SARS-CoV-2 are recommended to have 1 dose of the vaccine, thus they usually have both naturally acquired and vaccine-induced immunity. Thus, to reduce potential confounding we excluded residents who had a previous SARS-CoV-2 infection.

### Data Sources

Data were obtained through the Senior Shield surveillance program on demographics, results of the RT-PCR tests, COVID-19 hospitalizations, disease severity, COVID-19 deaths, and COVID-19 vaccination.

#### Primary End Point

SARS-CoV-2 infection was a dichotomous variable (yes or no). The incidence of SARS-CoV-2 infection was defined as a positive RT-PCR test, as documented in the Senior Shield system. RT-PCR screening policy for SARS-CoV-2 detection was unchanged throughout the study.

#### Additional End Points

COVID-19 hospitalizations and severity of illness (mild, moderate, severe) and COVID-19–related deaths were assessed. The management of COVID-19 and admission policy were not affected by vaccination status.

#### Follow-up

In 503 of 993 of the facilities, 50% or more of the residents were vaccinated within 5 days. The follow-up start date was more than 7 days after vaccination with the third BNT162b2 dose and for those who did not receive the booster dose, the follow-up start date was determined as more than 7 days after the midpoint of the 5 vaccination days in their facility. In facilities that did not reach vaccination coverage of 50% within 5 days, the follow-up start date was August 19, 2021, which corresponded to reaching 50% booster dose coverage among residents from all facilities. Vaccinated residents were included only if they were vaccinated within the 5-day window. This way we created a matched (common) calendar baseline for both study groups at the facility level (calendar weeks 32 to 35 in 2021). This was important given fluctuations in COVID-19 incidence during the study period. Both groups were followed until the earliest of the following events: SARS-CoV-2 infection, death due to reason other than COVID-19, receipt of the third dose of the vaccine in those who received only 2 doses, or end of follow-up on October 21, 2021. For the assessment of the additional end points, residents who tested positive for SARS-CoV-2 were monitored until hospitalization or death. The classification of disease severity was based on the documentation in the Senior Shield database.

#### The Independent Variable

COVID-19 booster (third dose) vaccination status was a dichotomous variable. The third dose group recipients vs recipients of 2 doses only, with the second vaccine dose given 5 months or earlier.

#### Covariates

The following variables were treated as confounders: age (years), sex, facility, population group (defined based on town of residence: general Jewish population, ultraorthodox Jewish population, or Arab population) and community-level socioeconomic status (SES).^22^ We also assessed the level of vaccination uptake of the third dose among all residents per facility.

### Statistical Analysis

Baseline characteristics of the study groups were described using means and SDs for continuous variables and counts, and percentages for categorical variables. Curves of cumulative incidence of SARS-CoV-2 infection among the study groups were created using Kaplan-Meier survival analysis and compared with the log-rank test. Cox proportional hazards regression models^23^ with follow-up time (days) as the time scale were constructed to estimate the hazard ratios (HRs) and 95% CIs for each end point, without multiplicity adjustment. The facilities were analyzed as strata (ie, the variable facility was treated as a fixed variable). Independent variables were COVID-19 third dose vaccination, sex, age, SES, population group, and calendar week. The proportional hazards assumption was tested using the Schoenfeld residuals, with no violations found. Multicollinearity was assessed using variance inflation factor with values less than 2 suggesting no multicollinearity

The following sensitivity analyses were performed: First, reanalysis of the data while considering the starting follow-up date as more than 14 days after vaccination with the third dose. Second, reanalyzing the data for overall SARS-CoV-2 infection separately for individuals who started the follow-up on weeks 32 to 33 and weeks 34 onward to further account for the frequent fluctuations in the disease incidence and uptake of the third dose in the country during the study period. Third, in a supplementary sensitivity analysis of all participants, we included the vaccination level of the facility as an independent variable, in addition of the participant's vaccination status, epidemiological week, age, sex, population group, and SES, and SARS-CoV-2 infection as the event of interest. Fourth, we assessed the robustness of the HRs from the main analysis to potential unmeasured confounders using the E-value,^24^ which is defined as the minimum strength of association on the risk ratio scale that an unmeasured confounder would need to have with both vaccination and the outcome to fully explain away the association between vaccination and study end points, conditional on the measured covariates. A large E-value suggests that substantial unmeasured confounding would be needed to explain away an effect (point) estimate, whereas a small E-value indicates that little unmeasured confounding would be sufficient to explain away an effect estimate.^24^

Missing data were low for all variables; therefore, we used complete case analysis. Two-sided *P* < .05 was considered statistically significant, without adjustment for multiplicity. Analyses were performed from August to October 2021 using SPSS Statistics for Windows version 27 (IBM Corp) and R software version 4.0.4 (R Project for Statistical Computing).

## Results

**Figure 1.**
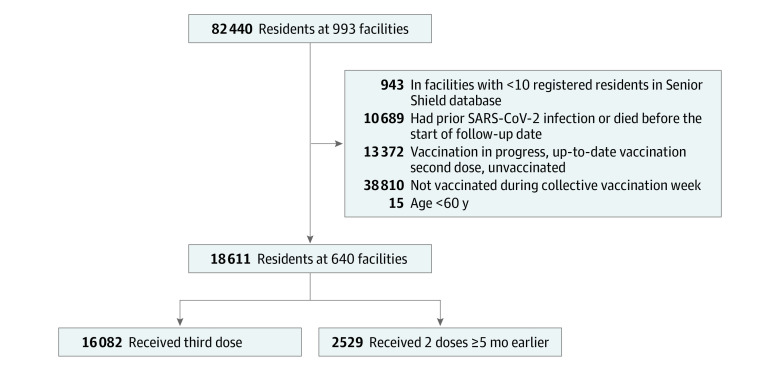
Flowchart of Selection of Participants

Of the 82 440 residents at 993 long-term care facilities registered in the Senior Shield database, 18 611 residents met the inclusion criteria at 640 facilities (Figure 1). Among those residents included in the analysis, 12 715 (68.3%) were female, 463 (2.5%) were from the Arab population, 16 976 (91.2%) were from the general Jewish population, and 618 (3.3%) were from the ultraorthodox Jewish population; the mean (SD) age was 81.1 (9.2) years; 16 082 were vaccinated with the third dose of BNT162b2 vaccine and 2529 received only 2 doses at least 5 months before the follow-up start date. The third recipients and the 2-dose–only vaccinees had comparable baseline characteristics, except that the third dose group was slightly older (mean [SD] age: 81.4 [9.0] years vs 79.3 [10.1] years) (Table 1).

**Table 1.** Baseline Characteristics of the Study Groups

Characteristic	Participants, No. (%)		
	2 Doses only (n = 2529)^a^	3 Doses (n = 16 082)^b^	Overall (N = 18 611)
Sex			
Female	1640 (64.8)	11 075 (68.9)	12 715 (68.3)
Male	884 (35.0)	4966 (30.9)	5850 (31.4)
Missing	5 (0.2)	41 (0.3)	46 (0.2)
Age, y			
Mean (SD)	79.3 (10.1)	81.4 (9.0)	81.1 (9.2)
Median (IQR)	80.0 (15.0)	83.0 (13.0)	83.0 (14.0)
Range	60-121	60-121	60-121
Community socioeconomic status rank			
Low	575 (22.7)	2912 (18.1)	3487 (18.7)
Medium	1084 (42.9)	5719 (35.6)	6803 (36.6)
High	734 (29.0)	6991 (43.5)	7725 (41.5)
Missing	136 (5.4)	460 (2.9)	596 (3.2)
Population group			
Arab population	130 (5.1)	333 (2.1)	463 (2.5)
General Jewish population	2120 (83.8)	14 856 (92.4)	16 976 (91.2)
Ultraorthodox Jewish population	159 (6.3)	459 (2.9)	618 (3.3)
Missing	120 (4.7)	434 (2.7)	554 (3.0)
Starting follow-up epidemiological week			
Mean (SD)	34.4 (0.9)	33.8 (0.8)	33.9 (0.9)
Median (IQR)	35.0 (1.0)	34.0 (1.0)	34.0 (2.0)
Range	32.0-37.0	32.0-36.0	32.0-37.0
Uptake of the third dose per facility, %			
0-59	1275 (50.4)	1902 (11.8)	3177 (17.1)
60-69	490 (19.4)	2988 (18.6)	3478 (18.7)
70-79	529 (20.9)	7620 (47.4)	8149 (43.8)
80-100	235 (9.3)	3572 (22.2)	3807 (20.5)
Facility type^c^			
Mental health	49 (1.9)	168 (1.0)	217 (1.2)
Geriatric facilities	1831 (72.4)	9766 (60.7)	11 597 (62.3)
Elder day care facilities	52 (2.1)	354 (2.2)	406 (2.2)
Welfare statutory accommodation (independent)	203 (8.0)	3758 (23.4)	3961 (21.3)
Welfare nursing homes (mentally frail)	113 (4.5)	856 (5.3)	969 (5.2)
Welfare disabilities	46 (1.8)	596 (3.7)	642 (3.4)
Other	235 (9.3)	584 (3.6)	819 (4.4)

^a^ Two doses of BNT162b2 vaccine at least 5 months before starting the follow-up date.

^b^ Received a third dose at least 5 months after vaccination with the second BNT162b2 vaccine dose.

^c^ The facilities that were classified as welfare disabilities include residents with physical or cognitive disabilities. The welfare nursing homes include residents that need some help and supervision in performing activities of daily living. Welfare statutory accommodation usually serves residents who are independent and capable of performing activities of daily living. Elder day-care facilities serve residents with variable functional status who live in their own homes, but they are transported to these facilities 2 to 3 times per week.

**Figure 2.**
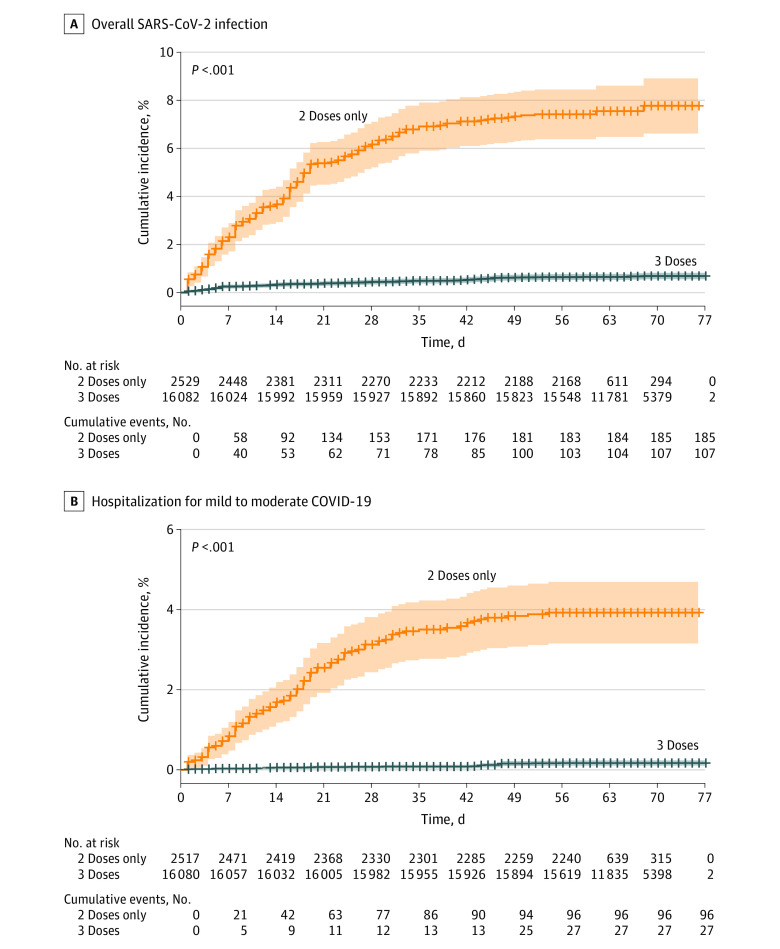
Cumulative Incidence of Overall SARS-CoV-2 Infection and Mild-to-Moderate COVID-19 Shaded areas represent 95% CIs and crosses on lines indicate censoring.

The median (IQR) follow-up duration was 66 (60-70) days among 3-dose recipients and 56 (53-62) days among 2-dose–only recipients. Overall, 107 residents had SARS-CoV-2 infection more than 7 days following vaccination with the booster dose, yielding a cumulative incidence of 0.7%; whereas 185 residents who received only 2 doses had SARS-CoV-2 infection, yielding a cumulative incidence of 7.5% (Figure 2A). The risk of SARS-CoV-2 infection was lower in the third-dose vaccinees compared with the 2-dose–only vaccinees (hazard ratio, 0.10 [95% CI, 0.07-0.15]). The adjusted HR (aHR) was 0.11 (95% CI, 0.07-0.14) **(**eTable 1 in the Supplement) against infection more than 7 days after vaccination with the third dose.

**Figure 3.**
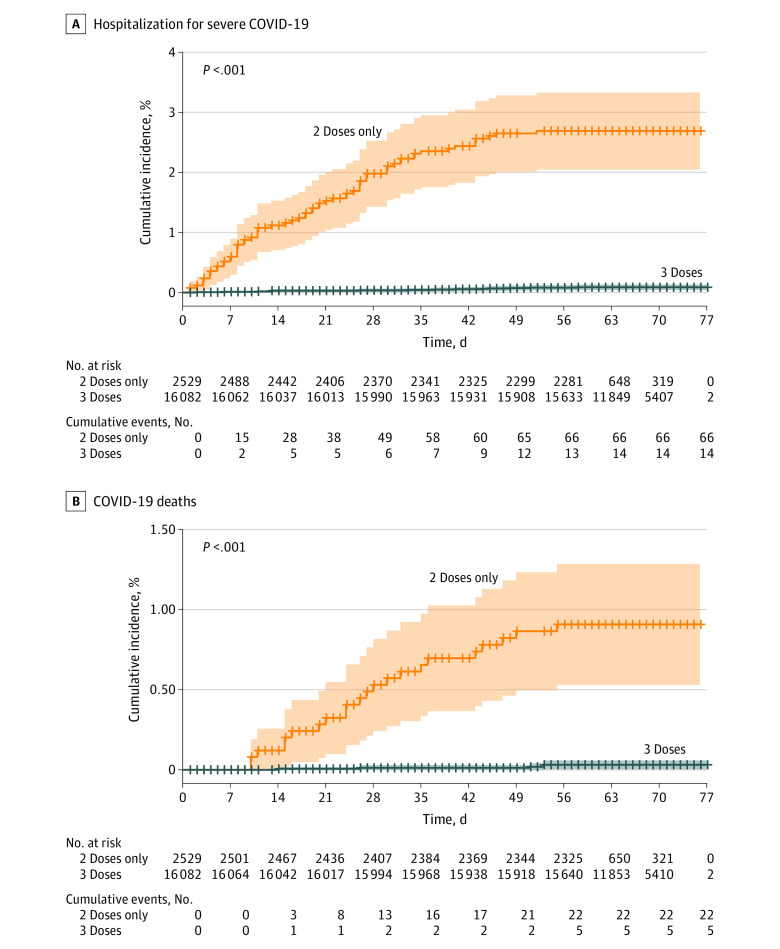
Cumulative Incidence of Severe COVID-19 Hospitalization and COVID-19–Related Death Shaded areas represent 95% CIs and crosses on lines indicate censoring.

The BNT162b2 third dose group had significantly lower risk of hospitalizations for COVID-19 (Figure 2B and Figure 3A) of mild-to-moderate severity (aHR, 0.07 [95% CI, 0.03-0.15) and severe illness (aHR 0.10 [95% CI, 0.04-0.24]) compared with those who received only 2 doses. Overall, 5 COVID-19–related deaths occurred among the 3-dose vaccinees during the follow-up period compared with 22 among the 2-doses–only vaccinees (cumulative rate, 0.04% vs 0.9%; aHR, 0.04 [95% CI, 0.009-0.16]) (Figure 3B; Table 2).

**Table 2.** Associations of BNT162b2 Third Dose Vaccination With SARS-CoV-2 Infection, COVID-19 Hospitalization, and Death More Than 7 Days Following Vaccination Among Residents of Long-term Care Facilities

End Point	No. of residents	No. of cases	Cumulative incidence %	Unadjusted HR (95% CI)^a^	*P* value^a^	Adjusted HR (95% CI)^b^	*P* value^b^
RT-PCR confirmed SASR-CoV-2 infection							
2 Doses of BNT162b2 vaccine^c^	2529	185	7.5	1 [Reference]	<.001	1 [Reference]	<.001
3 Doses of BNT162b2 vaccine	16 082	107	0.7	0.10 (0.07-0.15)		0.11 (0.07-0.15)	
Mild/moderate COVID-19 hospitalization							
2 Doses of BNT162b2 vaccine^c^	2517	96	3.9	1 [Reference]	<.001	1 [Reference]	<.001
3 Doses of BNT162b2 vaccine	16 080	27	0.2	0.08 (0.04-0.15)		0.07 (0.03-0.14)	
Severe COVID-19 hospitalization							
2 Doses of BNT162b2 vaccine^c^	2529	66	2.7	1 [Reference]	<.001	1 [Reference]	<.001
3 Doses of BNT162b2 vaccine	16 082	14	0.1	0.09 (0.04-0.22)		0.10 (0.04-0.24)	
COVID-19–related deaths^d^							
2 Doses of BNT162b2 vaccine^c^	2529	22	0.9	1 [Reference]	<.001	1 [Reference]	<.001^d^
3 Doses of BNT162b2 vaccine	16 082	5	0.04	0.03 (0.009-0.15)		0.04 (0.009-0.16)^d^	

Abbreviation: HR, hazard ratio.

^a^ Unadjusted Cox regression models.

^b^ Multivariable Cox regression model, adjusted for the variables age, sex, community-level socioeconomic status rank, population group, and epidemiological week.

^c^ The second dose was administered at least 5 months before the follow-up start date.

^d^ Multivariable Cox regression model, adjusted for the variables age, sex, and epidemiological week.

### Analysis of E-value

The E-value for the HR (and for the corresponding 95% CI) of the infection end point was 17.7 (12.8). The E-value for the HR (and for the corresponding 95% CI) was 28.06 (13.8) for hospitalization due to mild-to-moderate COVID-19, 19.5 (7.8) for severe disease, and 49.5 (11.9) for COVID-19–related deaths.

### Sensitivity Analysis

Reanalysis of the data while considering the follow-up start date as more than 14 days after vaccination with the third dose, showed significant inverse associations between vaccination with the third dose and each of the study end points (eTable 2 in the Supplement). Another sensitivity analysis that included individuals with follow-up start date on weeks 32 to 33 showed significantly lower risk for SARS-CoV-2 infection among the third dose group compared with the 2-dose–only group: aHR, 0.08 (95% CI, 0.04-0.16); *P* < .001. The respective results for those who started the follow-up on week 34 onward were aHR, 0.12 (95% CI, 0.07-0.19; *P* < .001).

An overall multivariable analysis that included the uptake of the third dose at the facility as an independent variable did not affect the association between the individual's booster vaccination with SARS-CoV-2 infection risk (aHR, 0.10 [95% CI, 0.08-0.14]; *P* < .001). This model also showed lower risk of SARS-CoV-2 infection: aHR was 0.42 (95% CI, 0.29-0.63; *P* < .001) for the third-dose coverage per facility of 60% to 69%, 0.50 (95% CI, 0.36-0.70; *P* < .001) for the third-dose coverage per facility of 70% to 79%, and 0.26 (95% CI, 0.15-0.43; *P* < .001) for the third-dose coverage per facility of 80% to100%, compared with 0% to 59%.

## Discussion

This cohort study found inverse associations between vaccination with the third dose of BNT162b2 with overall SARS-CoV-2 infection, COVID-19–related hospitalizations, severe illness, and COVID-19–related deaths among residents of LTCFs, compared with recipients of a 2-dose–only regimen at least 5 months prior to the study during the Delta variant surge in Israel. These results were already measurable following 7 days from vaccination, thus demonstrating the rapid reduction in the individual's risk of infection and disease in this vulnerable population. The inverse associations between vaccination with the third dose and the study end points were robust to potential unmeasured confounding as shown in our analysis of the E-value metric.

Studies from Israel have demonstrated effectiveness estimates of the BNT162b2 third dose within the range of 86% to 90% against infection,^21,25^ 92% to 97% against hospitalizations,^25,26^ and 92% to 95% against severe COVID-19,^21,26^ in the general population. The effectiveness of the third dose against death was estimated at 81% to 90%^26,27^ among relatively young population mostly above the age of 50 years. Our findings among LTCF residents corroborate these results. An analysis of reports to the Ministry of Health on adverse events showed similar rates of systemic events (eg, fever, fatigue) and local reactions (eg, pain, swelling, or redness in the injection site) after vaccination with the second and the third vaccine dose in the general population aged at least 60 years.^28^

Residents of LTCFs demonstrated blunted antibody responses after vaccination with 2 BNT162b2 doses compared with younger, nonfrail individuals.^29^ A study from Belgium on LTCF residents showed decays in humoral and cellular immune response 4 weeks following vaccination with 2 BNT162b2 doses, and the levels were generally lower compared with health care workers of the same facilities.^15^ In these reports older age correlated with lower antibody levels^29^ and decline in the humoral response, whereas diabetes and cancer were associated with decreased cellular immune response.^15^ A study from the United States demonstrated a relatively low vaccine effectiveness (74.7%) after 2 doses of mRNA COVID-19 vaccines among nursing homes residents before the Delta variant period, dropping to 53.1% during the Delta variant period.^30^

Humoral immune response to BNT162b2 resulted in high titer of neutralizing antibodies against the Alpha variant, but several times lower against the Delta variant.^31^ Thus, an important goal for the third administration was an increased antibody titer to improve the neutralization power and provide cross-protection against the Delta variant.

Our study provides new knowledge regarding the role of BNT162b2 third dose vaccination in preventing SARS-CoV-2 infection and COVID-19 hospitalizations and deaths among the older vulnerable population of LTCF residents. Vaccination policy targeting the prevention of symptomatic infection, severe disease, and deaths caused by SARS-CoV-2 is highly prioritized. Nonetheless, the prevention of overall SARS-CoV-2 infections, including asymptomatic infection also has public health merit, given the role of asymptomatic infections in the spread of the virus.^13^ Our study demonstrated that in the context of the frail population of LTCF residents, during the Delta variant surge, the third dose administration was essential to efficiently lower the risk of overall infection and not only severe disease, thus reducing the potential of the virus transmission in these facilities.

Our study has several strengths. We used data obtained in the framework of Senior Shield COVID-19 routine surveillance, in which SARS-CoV-2 PCR testing was performed systematically following predefined protocol and was not affected by vaccination status and assessed multiple end points. Vaccination was offered equally to all LTCFs across the country with high uptake.

### Limitations

Limitations of our study include the observational study design. In general, observational studies about vaccine effectiveness might be affected by selection bias and healthy user (vaccinee) effect. We did not have information on the reasons for opting not to receive the third dose of the vaccine. Information on comorbidity was also lacking, thus we could not assess whether the association between vaccination with the third dose and the study end points might differ among residents with certain comorbidities. Moreover, testing for SARS-CoV-2 might be influenced by vaccine receipt, thus introducing additional bias. However, in our study, testing policy for SARS-CoV-2 was unchanged throughout the study period and it was conducted according to predetermined screening protocols of Senior Shield task force.

## Conclusions

We found that LTCF residents who had received 3 doses of BNT162b2 vaccine had significantly lower risk for overall SARS-CoV-2 infection, COVID-19 hospitalizations, severe disease, and related deaths during the Delta variant surge in Israel compared with those who received 2 doses 5 months or longer prior to the potential exposure. These findings suggest that vaccination with the third dose of BNT162b2 was essential to the control of the Delta surge in the population of LTCFs.

## Notes

Supplement. **eTable 1.** Associations of BNT162b2 Vaccine Third Dose Vaccination and Other Co-Variates With SARS-CoV-2 Infection >7 Days Following Third Dose Administration **eTable 2.** Associations of BNT162b2 Third Dose Vaccination With SARS-CoV-2 Infection, COVID-19 Hospitalizations, and Deaths >14 Days Following Vaccination Among Residents of Long-Term Care Facilities
