# Supplementary material for: Association of BNT162b2 Vaccine Third Dose Receipt With Incidence of SARS-CoV-2 Infection, COVID-19–Related Hospitalization, and Death Among Residents of Long-term Care Facilities, August to October 2021
Source: JAMA Netw Open. 2022 Jul 1;5(7):e2219940. doi: 10.1001/jamanetworkopen.2022.19940 (PMC9250055; doi:10.1001/jamanetworkopen.2022.19940)
Supplement: Supplement. — eTable 1. Associations of BNT162b2 Vaccine Third Dose Vaccination and Other Co-Variates With SARS-CoV-2 Infection >7 Days Following Third Dose Administration eTable 2. Associations of BNT162b2 Third Dose Vaccination With SARS-CoV-2 Infection, COVID-19 Hospitalizations, and Deaths >14 Days Following Vaccination Among Residents of Long-Term Care Facilities [file jamanetwopen-e2219940-s001.pdf]

# Supplemental Online Content

Muhsen K, Maimon N, Mizrahi AY, et al. Association of BNT162b2 vaccine third dose receipt with incidence of SARS-CoV-2 infection, COVID-19–related hospitalization, and death among residents of long-term care facilities, August to October 2021. *JAMA Netw Open.* 2022;5(7):e2219940. doi:10.1001/jamanetworkopen.2022.19940

**eTable 1.** Associations of BNT162b2 Vaccine Third Dose Vaccination and Other Co-Variates With SARS-CoV-2 Infection >7 Days Following Third Dose Administration

**eTable 2.** Associations of BNT162b2 Third Dose Vaccination With SARS-CoV-2 Infection, COVID-19 Hospitalizations, and Deaths >14 Days Following Vaccination Among Residents of Long-Term Care Facilities

This supplemental material has been provided by the authors to give readers additional information about their work.

© 2022 Muhsen K et al. *JAMA Network Open.*

**eTable 1: Associations of BNT162b2 Vaccine Third Dose Vaccination and Other Co-Variates With SARS-CoV-2 Infection >7 Days Following Third Dose Administration**

| | Unadjusted HR (95% CI) [a] | P-value [a] | Adjusted HR (95% CI) [c] | P-value [c] |
|---|---|---|---|---|
| **BNT162b2 booster dose vaccination** | | <.001 | | <.001 |
| Two doses only [b] | 1.00 | | 1.00 | |
| third dose vaccination > 7 days | 0.10 (0.07, 0.15) | | 0.11 (0.07, 0.15) | |
| **Age, years** | 1.00 (0.98, 1.01) | 0.65 | 1.00 (0.98, 1.02) | 0.99 |
| **Sex** | | 0.18 | | 0.05 |
| Female | 1.00 | | 1.00 | |
| Male | 1.18 (0.92, 1.52) | | 1.29 (0.99, 1.68) | |
| **Residential socioeconomic status** | | 0.87 | | 0.72 |
| Low | 1.00 | | 1.00 | |
| Intermediate | 1.05 (0.69, 1.59) | 0.81 | 1.07 (0.67, 1.71) | 0.76 |
| High | 0.96 (0.60, 1.51) | 0.86 | 0.93 (0.56, 1.55) | 0.78 |
| **Population group** | | 0.76 | | 0.87 |
| Arab population | 1.00 | | 1.00 | |
| General Jewish population | 0.97 (0.33, 2.84) | 0.69 | 1.10 (0.38, 3.16) | 0.85 |
| Ultraorthodox Jewish population | 1.21 (0.59, 2.51) | 0.59 | 1.21 (0.57, 2.55) | 0.61 |
| **Calendar epidemiological week** | 0.10 (0.04, 0.28) | <.001 | 0.34 (0.12, 1.00) | 0.05 |

HR: Hazard ratio; CI: Confidence intervals

[a] Unadjusted Cox regression models

[b] The second dose was administered ≥5 months before the follow-up starting date

[c] Multivariable Cox regression model, adjusted for the variables in the table and epidemiological week

© 2022 Muhsen K et al. *JAMA Network Open.*

**eTable 2: Associations of BNT162b2 Third Dose Vaccination With SARS-CoV-2 Infection, COVID-19 Hospitalizations, and Deaths >14 Days Following Vaccination Among Residents of Long-Term Care Facilities**

| | Number of residents | Number of cases | Cumulative incidence | Unadjusted HR (95% CI) [b] | P-value [b] | Adjusted HR (95% CI) [c] | P-value [c] |
|---|---|---|---|---|---|---|---|
| **RT-PCR confirmed SASR-CoV-2 infection** | | | | | <.001 | | <.001 |
| Two doses of BNT162b2 vaccine [a] | 2440 | 127 | 5.6% | 1.00 | | 1.00 | |
| Three doses of BNT162b2 vaccine | 16,020 | 67 | 0.4% | 0.08 (0.05, 0.13) | | 0.09 (0.05, 0.14) | |
| **Mild/moderate COVID-19 hospitalization** | | | | | <.001 | | <.001 |
| Two doses of BNT162b2 vaccine [a] | 2431 | 68 | 2.9% | 1.00 | | 1.00 | |
| Three doses of BNT162b2 vaccine | 16,019 | 19 | 0.1% | 0.06 (0.03, 0.12) | | 0.05 (0.02, 0.11) | |
| **Severe COVID-19 hospitalization** | | | | | <.001 | | <.001 |
| Two doses of BNT162b2 vaccine [a] | 2440 | 44 | 1.9% | 1.00 | | 1.00 | |
| Three doses of BNT162b2 vaccine | 16,020 | 9 | 0.1% | 0.06 (0.02, 0.16) | | 0.06 (0.02, 0.17) | |
| **COVID-19 related deaths [d]** | | | | | <.001 | | <.001 [d] |
| Two doses of BNT162b2 vaccine [a] | 2440 | 14 | 0.6% | 1.00 | | 1.00 | |
| Three doses of BNT162b2 vaccine | 16,020 | 4 | 0.02% | 0.04 (0.007, 0.19) | | 0.03 (0.006, 0.20) [d] | |

[a] The second dose was administered ≥5 months before the follow-up start date
[b] Unadjusted Cox regression models
[c] Multivariable Cox regression model, adjusted for the variables age, sex, community-level socioeconomic status rank, population group, and epidemiological week
[d] Multivariable Cox regression model, adjusted for the variables age, sex, and epidemiological week
HR: hazard ratio; CI: confidence intervals

© 2022 Muhsen K et al. *JAMA Network Open.*
